# Outstanding Ultra‐Low Freezing Tolerance in Moss Species: Insights From Recovery Ability

**DOI:** 10.1002/pei3.70081

**Published:** 2025-09-24

**Authors:** Surayya Mustapha Muhammad, Wenwan Bai, Ruirui Yang, Haron Salih, Xiujin Liu, Yuqing Liang, Dina Mahesati, Daoyuan Zhang, Xiaoshuang Li

**Affiliations:** ^1^ State Key Laboratory of Desert and Oasis Ecology, Key Laboratory of Ecological Safety and Sustainable Development in Arid Lands, Xinjiang Institute of Ecology and Geography Chinese Academy of Sciences Urumqi China; ^2^ University of Chinese Academy of Sciences Beijing China; ^3^ Xinjiang Key Laboratory of Conservation and Utilization of Plant Gene Resources, Xinjiang Institute of Ecology and Geography Chinese Academy of Sciences Urumqi China

**Keywords:** age, freezing stress, moss, protonema, recovery rate, relative water content

## Abstract

Freezing temperature is a key environmental factor that influences plant growth and distribution. Mosses exhibit remarkable resistance to freezing stress due to their unique morphological and physiological traits. The protonema, which is the initial structure formed during the germination of a moss spore, exhibits a short life cycle and is highly sensitive to environmental changes. In this study, the protonemas of three moss species, *Physcomitrium patens*, *Bryum argenteum* , and *Syntrichia caninervis* , were harvested when they were 5, 10, and 15 days old. Protonemas were air dried for 0, 1, 2, and 12 h. Air‐dried protonemas were kept at −80°C for 6 months to evaluate their resilience to ultra‐low freezing stress. This resilience was assessed at 6, 12, and 18 days after re‐culture. The three moss species exhibited varying degrees of freezing tolerance. *P. patens* did not recover after −80°C treatment, fully dried 10‐days‐old *B. argenteum* achieved highest recovery rate of 99.6% ± 0.2% while fully dried 5‐days‐old *S. caninervis* achieved the highest recovery rate of 98.6% ± 0.5%. The regeneration rate was influenced by both relative water content (RWC) and age. An analysis using a linear mixed‐effects model indicated that the impact of RWC (effect size = 0.75) was greater than that of age (effect size = 0.35). This research provides valuable insights into the resilience of moss protonemas after exposure to −80°C, emphasizing the importance of protonema in abiotic stress research. These findings are crucial for developing methods to preserve and maintain terrestrial ecosystems in arid regions.

## 1 Introduction

Temperature extremes, particularly freezing temperatures, pose significant challenges to evergreen species that inhabit frost‐prone regions (Lenné et al. 2010). Freezing stress is a critical factor that limits plant growth and geographical distribution because of the lack of liquid water due to the formation and accumulation of ice crystals in the apoplast, resulting in the severe dehydration of plant cells and disrupting their homeostasis (Ouellet and Charron 2013; Juurakko et al. 2021). Plants have evolved several adaptive mechanisms to mitigate the harmful effects of cell damage and injury caused by freezing stress, including changes to their physiology, metabolism, and cell structure (Qari et al. 2022).

Whereas bryophytes are characterized by a dominant gametophyte generation, tracheophytes have a dominant sporophyte generation, and adaptations to their life cycle have allowed them to grow in terrestrial environments, ranging from the tropics to Antarctica, for over 400 million years (Proctor et al. 2007; Shaw et al. 2011; Hisanaga et al. 2019). The successful adaptation of bryophytes to terrestrial environments is partly attributed to their ability to acquire tolerance to freezing stress, which varies among species (La Farge et al. 2013), but can also depend on age, tissue, natural habitat, and season (Agurla et al. 2018). Mosses can endure freezing stress partially due to their unique morphological features, physiological responses, and biochemistry (Perroud et al. 2018; Tanaka et al. 2018; Kearly 2024). Studies on Antarctic mosses, including *Grimmia antarctici*, revealed their remarkable resilience to extreme environmental conditions, particularly low temperatures and freeze–thaw cycles. These plants employ photoinhibition as a protective mechanism by down‐regulating photosystem II to prevent damage, allowing them to recover under favorable conditions. During that process, two xanthophyll cycle pigments, zeaxanthin and antheraxanthin, play a crucial role in protecting photosystem II from photo‐inhibitory damage. This series of adaptations, combined with desiccation tolerance, enables Antarctic mosses to thrive in harsh environments and may enhance their reproductive success (Lovelock, Jackson, et al. 1995; Lovelock, Osmond, and Seppelt 1995; Perera‐Castro et al. 2021). Ochi (1952) reported the tolerance of 18 moss species in various locations to freezing (−20°C) stress. Some mosses have even demonstrated resistance to freezing stress at −27°C (Lenné et al. 2010). The remarkable ability of bryophytes to adapt to diverse environmental conditions, combined with their widespread global distribution, makes them an ideal model for studying the impact of environmental stressors (Ladrón De Guevara and Maestre 2022; Oliver et al. 2000).

A model moss, *Physcomitrium patens*, is widely distributed in temperate zones and thrives near lakes and rivers, and in damp open‐ground environments (Cove 2005; Frank et al. 2005). *P. patens* has been used in research as a model moss due to its remarkable characteristics, including its stress tolerance and high homologous frequency of recombination (Hernandez‐Coronado et al. 2016; Perroud et al. 2018; Bi et al. 2024). *Bryum argenteum* , a versatile moss that is distributed virtually throughout the world (Huttunen et al. 2018), has recently emerged as a model moss for studying and identifying the molecular mechanism underlying potential stress‐related genes in response to abiotic stresses (Gao et al. 2015; Liang et al. 2021). In Northwestern China, *B. argenteum* is one of the organisms that inhabit the soil and plays an important role in maintaining ecosystems where temperatures tend to be low (Zhang et al. 2007; Gao et al. 2017). *Syntrichia caninervis* is widely distributed in cold deserts across central Asia and North America (Proctor et al. 2007; Coe et al. 2021). In the Gurbantunggut Desert in China, *S. caninervis* is the dominant moss species that grows on the soil surface (Zhang et al. 2006). Recently, *S. caninervis* emerged as a valuable model organism for research, owing to its exceptional resistance to various environmental stressors, including desiccation and extremely low temperatures (Gao et al. 2014, 2025; Silva et al. 2021; Li et al. 2024).

Despite significant advances in understanding the resistance of mosses to freezing conditions (Oliver et al. 2000; Minami et al. 2003; Lenné et al. 2010; Tanaka et al. 2018; Silva et al. 2021; Li et al. 2024), it remains unclear whether protonema can survive ultra‐low temperature freezing for prolonged periods. In this study, uniform samples, which were matched with respect to age and relative water content (RWC) derived from the tissue cultures of three moss species, *P. patens* , *B. argenteum* , and *S. caninervis*, were used to investigate their responses and tolerance to treatment at −80°C. The objective was to identify key factors influencing their survival strategies and recovery abilities after ultra‐low temperature treatment. The most important finding was that RWC and age are key factors that influence the resilience of these moss species to ultra‐low temperatures, conferring on them freezing tolerance. The recovery rates of these mosses further illustrate their capacity to adapt to extreme low‐temperature stress. These results highlight the importance of specific survival strategies by these mosses in response to exposure to ultra‐low temperatures and provide a foundation for understanding the freezing tolerance in protonema. The results also provides valuable insight into preservation strategies and future research on the molecular mechanism of stress responses in moss species.

## 2 Materials and Methods

### 2.1 Culture Conditions of Moss Protonemas

The protonemas of *P. patens* , *B. argenteum* , and *S. caninervis* used in this study were obtained from our laboratory at the Xinjiang Institute of Ecology and Geography, University of Chinese Academy of Sciences. Knop's basal medium, which included 8 g/L agar and 0.75 g/L ammonium tartrate (pH 5.8) was autoclaved at 121°C for 20 min (Reski and Abel 1985), then poured into Petri dishes (diameter 90 mm). Protonemas (1.5 g from each moss species) were removed from dishes, added to a 50 mL test tube, and homogenized in 30 mL of distilled deionized water using a high‐speed dispersion (T18, IKA, Staufen, Germany) homogenizer. One milliliter of the protonema suspension was cultured on Knop's medium. The *P. patens* protonemas were cultured on Knop's medium at 25°C under a 16‐h photoperiod with a photosynthetic photon flux density (PPFD) of 55 μmol m^−2^ s^−1^ (Frank et al. 2005). The protonemas of *S. caninervis* and *B. argenteum* were cultured on Knop's medium at 22°C under a 16‐h photocycle with a PPFD of 80–100 μmol m^−2^ s^−1^ (Coe et al. 2021) and placed in growth chambers (BIC‐400, Shanghai Boxun Medical Biological Instrument for Crops, Shanghai, China). Images of the growth of protonemas were captured with a BX51 light microscope (Olympus, Tokyo, Japan) at 0, 1, 3, 5, 7, 10, 11, 13, 15, 20, and 30 days after the start of culture. Chlorophyll (Chl) content of all three moss species was measured at 5, 10, 15, and 30 days after the start of culture. Each stage included three biological replicates and 30 Petri dishes. Using photographed images, the growth area of protonemas was calculated using ImageJ analysis software (The National Institutes of Health, Bethesda, MD, USA) (Stark and McLetchie 2006) at various time points (3, 5, 7, 10, 11, 13, 15, 20, and 30 days).

### 2.2 Chlorophyll Content of Moss Protonemas

Chl content was spectrophotometrically determined using a spectrophotometer (Biomate 3S, Thermo Fisher Scientific, Waltham, MA, USA), following the methods described by Lichtenthaler and Wellburn (1983). A total of 0.1 g of *B. argenteum* , *P. patens* , and *S. caninervis* protonemas that were 5, 10, 15, and 30 days old were removed from Petri dishes to measure Chl content. Pigments were extracted by incubating the protonemas in 2 mL of 96% ethanol at room temperature (RT) (25°C) for 12 h in the dark. The extracts were centrifuged at 10,000 rpm for 2 min, and the supernatants were used to measure absorbance (A) at wavelengths of 649, and 665 nm. Chlorophyll *a*, *b* (Chl *a*, Chl *b*) and total Chl were calculated using the following equations (Lichtenthaler and Wellburn 1983): Chl *a* (mg/g) = 13.95 × (A)_665_–6.88 × (A)_649_, Chl *b* (mg/g) = 24.96 × (A)_649_–7.32 × (A)_665_, and total Chl content (mg/g) = Chl *a* + Chl *b*. All measurements of Chl content were made as three biological replicates.

### 2.3 Freezing‐Drying Stress Treatments

Protonemas of the three moss species were removed from Petri dishes at distinct developmental stages (5, 10, and 15 days) and exposed to controlled dehydration treatments. RWC was assessed by air‐drying the protonemas (fully hydrated 0.2 g) at RT and 30%–40% relative humidity (RH) for 0 (control), 1, 2, and 12 h in a sterile lamina airflow (SW‐CJ‐2FD, Suzhou Antai Airtech, Suzhou, China) to achieve fresh weight. Dry weight was assessed after oven‐drying the protonemas at 55°C for 48 h.

RWC was calculated as follows: (Wx − DW)/(W0 − DW) × 100, where Wx is the weight of protonemas after air‐drying, DW is the weight of protonemas after oven drying, and W0 is the initial weight of protonemas after they were removed from Petri dishes.

**FIGURE 1:**
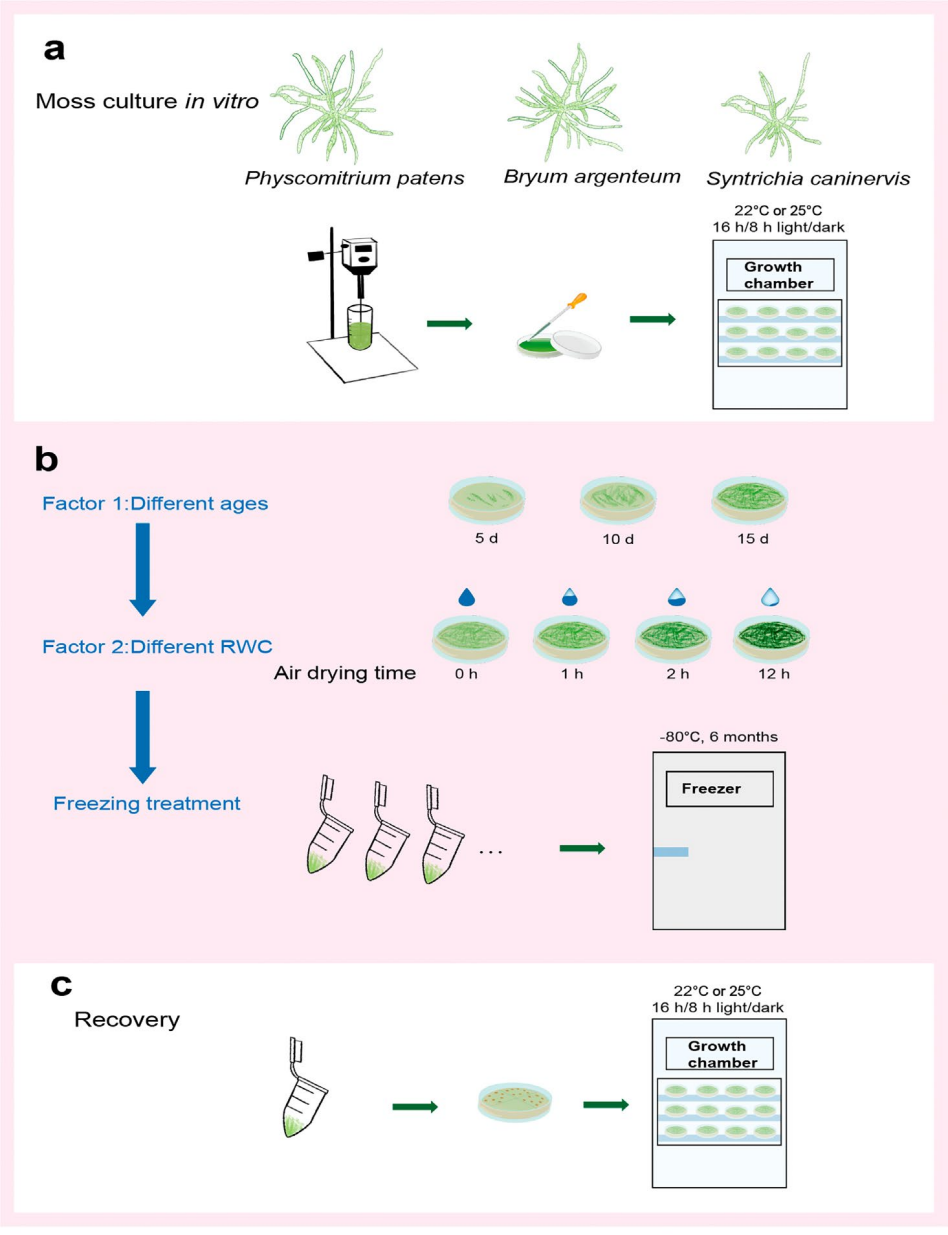
Diagram illustrating the growth of protonemas, ultra‐low temperature treatment, and the recovery process of three moss protonemas. (a) In vitro growth and culture under controlled conditions on Knop's medium. (b) The protonemas of three ages (5, 10, and 15 days) were air dried for 0 (control), 1, 2, and 12 h in a sterile lamina airflow at RT, then exposed to extreme low‐temperature stress. Factor 1: Different ages (5, 10, and 15 days) of protonemas. Factor 2: Air‐drying protonemas at 0, 1, 2, and 12 h, then storing them at −80°C for 6 months. (c) Thawed protonemas were grown on fresh Knop's medium to examine their ability to recover from ultra‐low temperature freezing stress.

Dried protonemas were stored directly at −80°C in an ultra‐low temperature freezer (TDE 40086FV‐ULTS, Thermo Fisher Scientific) for 6 months (Figure 1). After this storage period, the protonemas of the three moss species were placed at RT for 10 min to thaw, transferred onto fresh Knop's medium in Petri dishes, and cultured in a growth chamber under the growth conditions described earlier (Figure 1 and Table S1). To assess the effects of freezing stress, photos were taken at 0, 6,12 and 18 days to monitor the recovery, and the recovery rate of protonemas was calculated based on the following formula (Guo and Zhao 2018):

$$\text{Regenration rate}\;\left(\%\right)=\frac{\text{Number of regenerated protonema clumps}}{\text{Total number of protonema clumps}}\times 100$$

The effects of RWC and age on the recovery rate of protonemas were determined using a linear mixed‐effect model (Wu et al. 2022).

### 2.4 Statistical Analysis

Data are presented as the mean ± standard deviation (SD) derived from three biological replicates. Data were analyzed using two‐way analysis of variance (ANOVA) at a 95% confidence level (CL). Significant differences were determined using Fisher's least significant difference (LSD) multiple comparison test. Statistical significance relative to the control group was assessed at *p < 0.05, **p < 0.01, and ***p < 0.001. Column graphs were generated using GraphPad Prism 9 software (version 9.0.0, 2020) and images were processed in Adobe Photoshop CS6 (version 13.0, 2024).

## 3 Results

### 3.1 Growth and Chlorophyll Content of Protonemas

**FIGURE 2:**
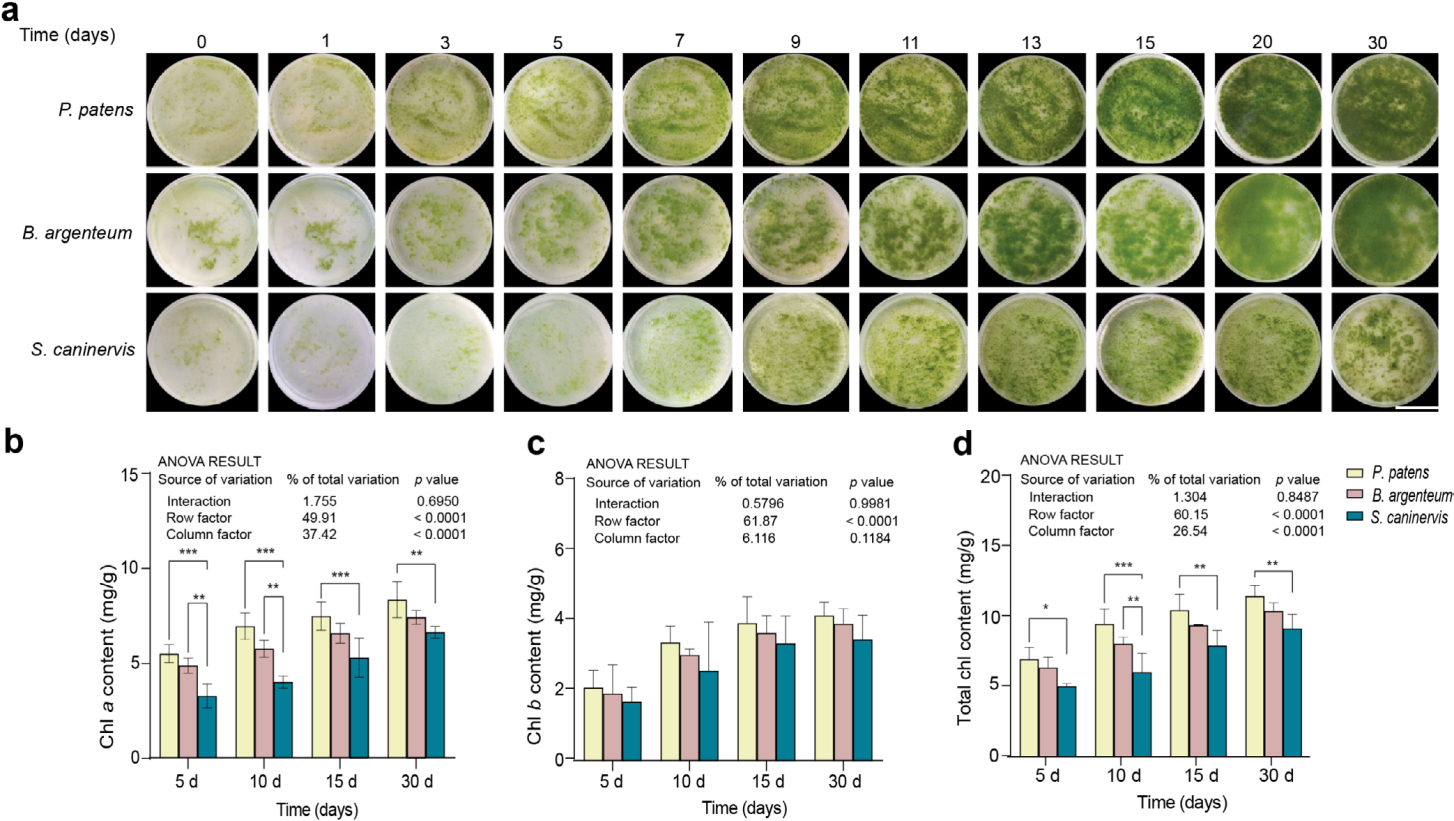
Morphological and physiological responses of the protonemas of three moss species after different days of culture on Knop's basal medium. (a) Phenotypic response of protonemas at various time points over the 30‐day culture period. (b) Chl *a*, (c) Chl *b*, and (d) total chlorophyll content of *P. patens* , *B. argenteum* , and *S. caninervis* protonemas measured at 5, 10, 15, and 30 culture days. Chlorophyll was extracted by incubating the protonemas in 2 mL of 96% ethanol (25°C) for 12 h in the dark. The extracts were centrifuged at 10,000 rpm for 2 min, and the supernatants were used to quantify Chl *a*, Chl *b*, and total Chl using measurements at wavelengths of 649 nm, and 665 nm, respectively. The columns are color‐coded to represent different moss species. Data were analyzed using two‐way ANOVA at a 95% CL. Significant differences within group were determined using Fisher's LSD multiple comparison test. The data are expressed as the mean ± SD from three biological replicates. The ANOVA result represent two factors, column represent different specie and row represent age. *p < 0.05, **p < 0.01, and ***p < 0.001.

The primary objective of these measurements was to evaluate the typical growth of three moss species, *P. patens* , *B. argenteum* , and *S. caninervis* , under conventional in vitro culture conditions. An assessment of the growth of protonemas on Petri dishes revealed that *P. patens* grew the fastest, becoming visibly green at 5 days and nearly covering the entire surface of the medium in Petri dishes by day 13. A vibrant green color was maintained until 20 days before becoming darker at 30 days. *B. argenteum* protonemas grew at a moderate rate, developing green patches at 5–7 days and covering the entire surface of the medium in Petri dishes by 15–20 days, with relatively uniform coloration up to 30 days. *S. caninervis* grew the slowest and showed modest greening between 9 and 13 days and sparse coverage of the surface of the medium in Petri dishes by 30 days (Figure 2a). The development of protonemas in all three species began with a small, single filament, as observed under a microscope at day 0, and this was designated at the start of culture (Figure S1a). After 1 day of culture, the protonemas of all three species formed buds that displayed an aggregated growth pattern (Figure S1). The number of buds produced was species‐dependent, with *P. patens* (10.8 ± 7.4) forming the most, followed by *B. argenteum* (9.2 ± 6.8), then *S. caninervis* (7.8 ± 6.1) (Figure S2c and Table S5). Similarly, the growth area of protonemas expanded over time, with *P. patens* ((2.2 ± 0.9) × 10^6^ mm^2^) displaying the largest growth area, followed by *B. argenteum* ((1.9 ± 0.9) × 10^6^ mm^2^), then *S. caninervis* ((1.7 ± 0) × 10^6^ mm^2^) (Figure S1a,b and Table S5). The Chl content in protonemas also increased over time: *P. patens* had the highest level of Chl *a* and total Chl content (13.6 ± 2.6 mg/g), followed by *B. argenteum* (11.7 ± 2.4 mg/g), while *S. caninervis* (9.6 ± 2.5 mg/g) had the lowest total Chl content (Figure 2b–d and Table S5). There were significant differences in Chl *a* and total Chl contents among the three moss species, but no significant differences were found in Chl *b* content (Figure 2b–d and Table S5). Based on the phenotypic observations and contents of Chl, the 5‐day (early stage), 10‐day (growth phase) and 15‐day growth stages represent the three most pronounced periods of growth and development. Therefore, moss protonemas at these three growth stages were selected for subsequent RWC measurements and freezing recovery experiments.

### 3.2 Relative Water Content of Moss protonemas

**FIGURE 3:**
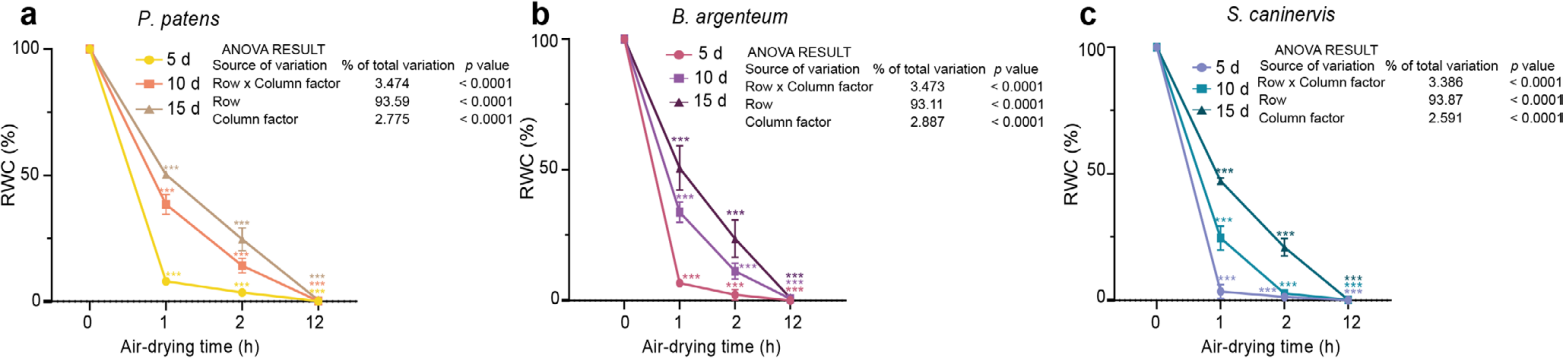
Changes in RWC of the protonemas of three moss species at different air‐drying time points across three ages (5, 10, and 15 days). RWC of the protonemas was measured after 0, 1, 2, and 12 h of air‐drying at room temperature (25°C) and 30%–40% relative humidity in a sterile laminar airflow cabinet to achieve fresh weight. Dry weight was determined after drying protonemas in an oven at 55°C for 48 h. (a) *P. patens* , (b) *B. argenteum* , and (c) *S. caninervis* . Data were analyzed using two‐way ANOVA at a 95% CL. Significant differences compared to the control (0 h) were determined using Fisher's LSD multiple comparison test. The data are expressed as the mean ± SD from three biological replicates. The ANOVA result represent two factors, column represent different protonema age and row represent air‐drying time. The ages of the protonemas are indicated by three shapes: circles, rectangles, and triangles for 5, 10, and 15 days, respectively. ***p < 0.001.

In the three moss species, there was a significant effect of protonema age on the dehydration kinetics. RWC decreased faster in the younger protonemas of all three moss species. In the young (5‐day‐old) protonemas of the three moss species, RWC rapidly decreased by over 92% (from 3.49% to 8.0%) after 1 h of air‐drying, reaching 96% (ranging from 0.9% to 3.5%) after 2 h, and approaching zero (ranging from 0% to 0.3%) after 12 h. In the 10‐day‐old protonemas of *P. patens* , *B. argenteum* , and *S. caninervis* , RWC decreased by more than 65% (ranging from 11.21% to 24.5%) after 1 h of air‐drying, reached 85% (ranging from 2.69% to 14.20%) after 2 h, then became nearly zero (ranging from 0% to 0.3%) after 12 h. In 15‐day‐old protonemas, RWC declined significantly by 46% (ranging from 47% to 53%) after 1 h of air‐drying and continued to decrease by 65% (ranging from 20% to 25%) and became less than 0.5% (ranging from 0.1% to 0.5%) after 2 and 12 h of air‐drying, respectively (Figure 3 and Table S2).

### 3.3 Freezing Tolerance of *P. patens*

**FIGURE 4:**
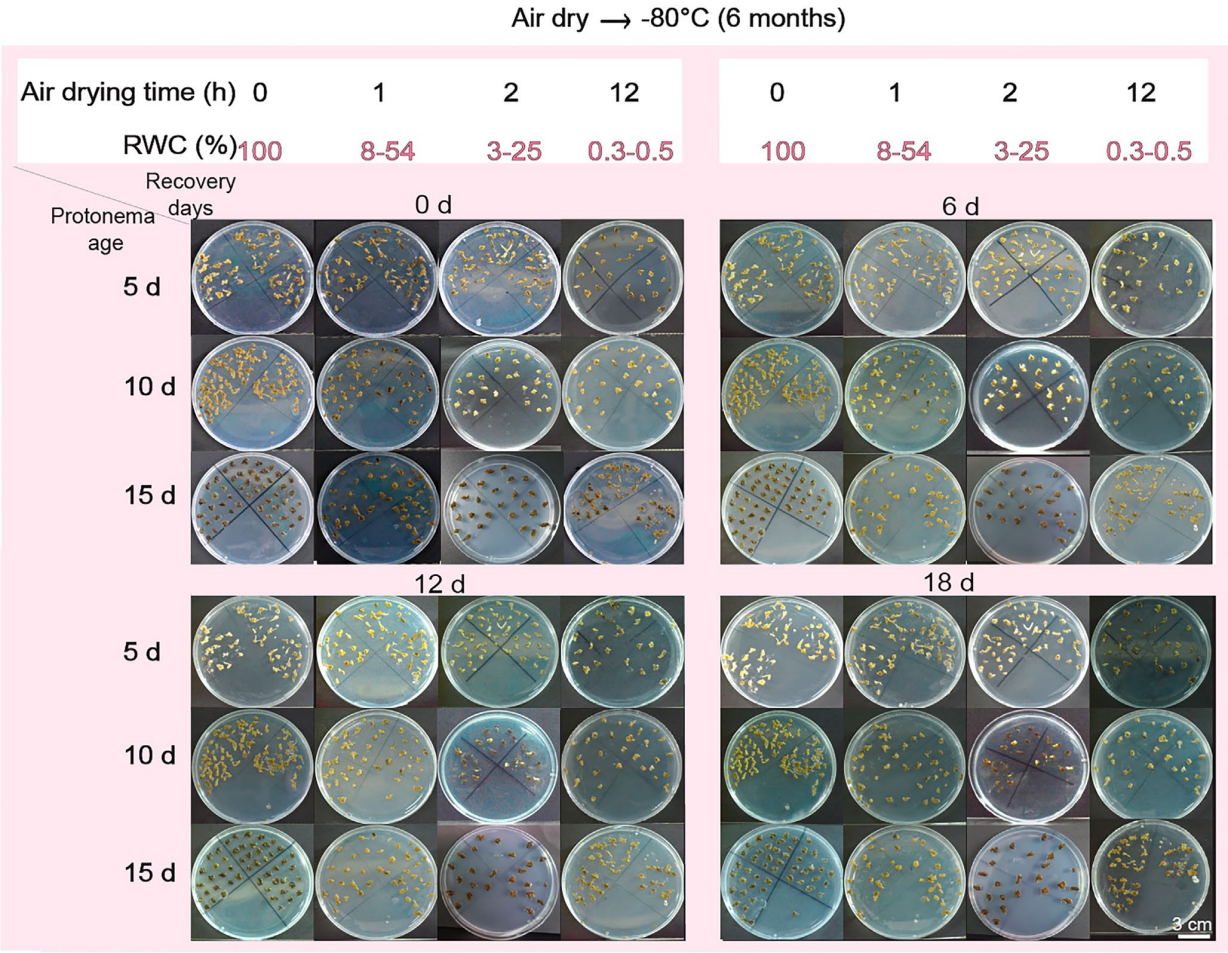
Phenotypic recovery of *P. patens* protonemas on Knop's medium after freezing at ultra‐low temperatures. Protonemas of different ages (5, 10, and 15 days) were air dried for 0, 1, 2, and 12 h (0 h served as the control) and then stored at −80°C in an ultra‐low temperature freezer for 6 months. After thawing, the stored *P. patens* protonemas were transferred onto fresh Knop's medium on Petri dishes and incubated in a growth chamber. The phenotypes of protonemas were observed at 0, 6, 12, and 18 days of recovery.

The effect of freezing stress on the recovery rate of *P. patens* protonemas was assessed at different recovery days (0, 6, 12, and 18 days). All dehydration treatments and ages responded in the same way, resulting in a loss of physiological functionality, as evidenced by their lack of growth after recovery. At all the monitoring days, neither regeneration nor recovery was observed in any age group (5, 10, and 15 days of age) (Figure 4). These results indicate that *P. patens* protonemas lost their ability to recover after exposure to ultra‐low temperature.

### 3.4 Relative Water Content and Age: Impact on Freezing Tolerance of *B. argenteum*

**FIGURE 5:**
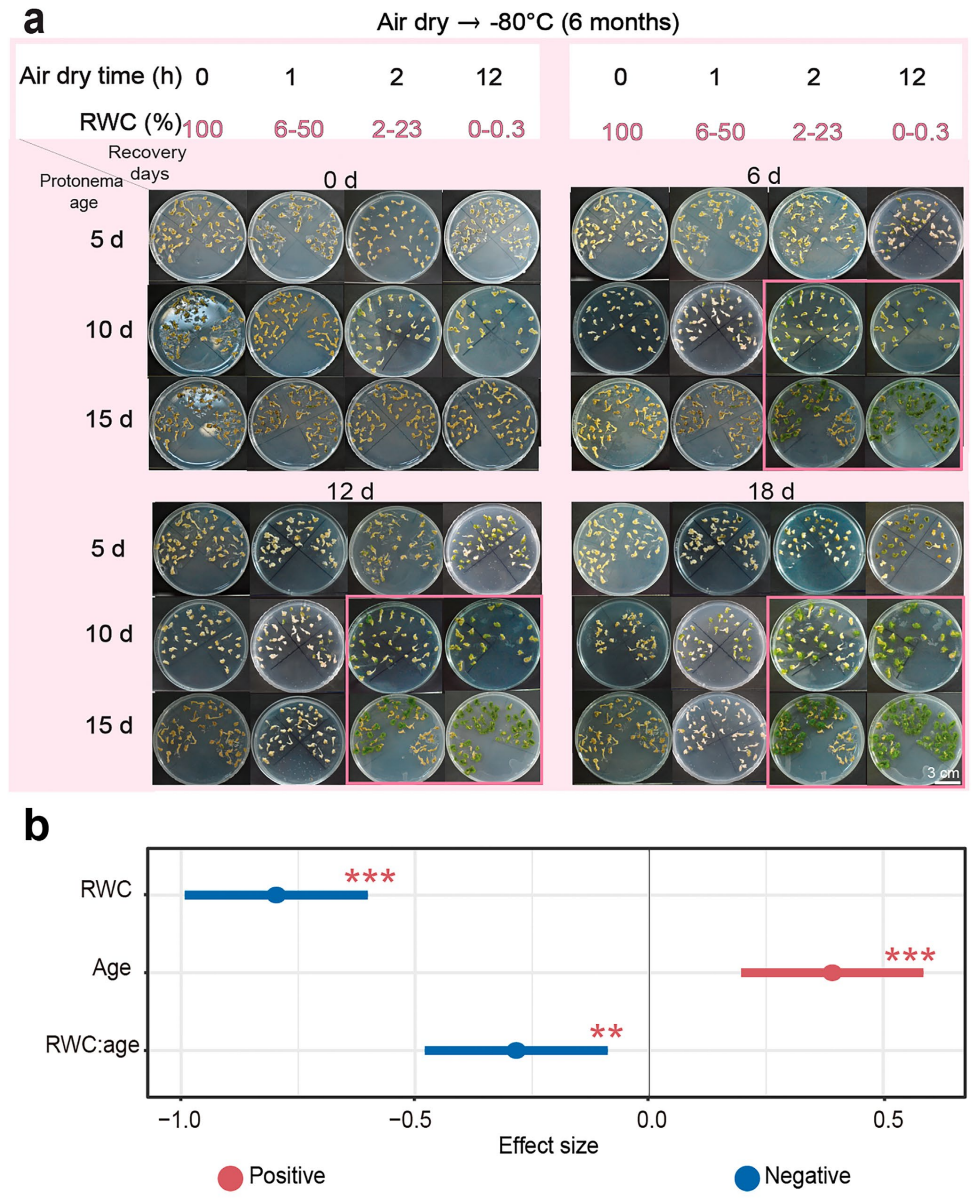
Growth recovery of *B. argenteum* protonemas on Knop's medium after freezing at an ultra‐low temperature. (a) In vitro growth of protonemas on Petri dishes after different air‐drying times, and of three ages. (b) Effect of RWC, age, and RWC and age on the recovery rate of protonemas using a linear mixed‐effects model. The recovery rate of protonemas was measured at 6‐day intervals starting from the day of culture. Protonemas were collected when they were 5, 10, and 15 days old and exposed to air‐drying for 0, 1, 2, and 12 h. Dried protonemas were then stored at −80°C in an ultra‐low temperature freezer for 6 months. After storage, the protonemas were transferred to fresh Knop's medium in Petri dishes and cultured in a growth chamber. Phenotypic observations were made at 0, 6, 12 and 18 days after recovery. Pink boxes highlight the highest recovery rates in response to freezing stress at −80°C. Data are presented as the mean ± SD of the estimated effect size. Statistical significance: **p < 0.01, and ***p < 0.001.

The protonemas of *B. argenteum* displayed significant variation in recovery rate depending on RWC (time of air‐drying: 0, 1, 2, and 12 h), age (5, 10, and 15 days old) and days of recovery (0, 6, 12, and 18 days) (Figure 5 and Table S3). The results show that RWC was the main factor influencing the recovery rate of *B. argenteum* protonemas in response to freezing at an ultra‐low temperature. Fully hydrated (100% RWC) protonemas did not recover (i.e., zero = no recovery rate) at any age, but when RWC dropped, the recovery rate of the protonemas increased. After 1 h of air drying (8.0% RWC), 5 days old protonemas showed a 2.7% recovery rate, but after 2 h (2.2% RWC) and 12 h of air drying (0% RWC), the recovery rate increased to 23.6% and 80.5%, respectively. After 1 h of air drying (33.78% RWC), the recovery rate was 33.4% in 10‐day‐old protonemas, but after 2 h of air drying (11.21% RWC), the recovery rate increased to 59.23%, while the highest recovery rate (100%) was recorded after 12 h of air drying (0% RWC). After 1 h of air drying (50.6% RWC), 15‐day‐old protonemas displayed the lowest recovery rate (23.06%) followed by 2 h of air drying (23.6% RWC) with an 83.86% recovery rate, while the highest recovery rate (94.4%) was recorded after 12 h of air drying (0.3% RWC) (Figure S2). The linear mixed‐effects model was employed to assess the impact of RWC, age, and its interaction on regeneration rate, with the effect size indicating the direction and magnitude of the treatment effect. A comparison of the effect values shows that for *B. argenteum* protonemas, RWC (effect size = −0.80) had a negative effect on regeneration rate, and its influence on regeneration rate was greater than that of age (effect size = 0.39) (Figure 5b). The response of *B. argenteum* protonemas to freezing stress was significantly influenced by RWC. RWC influenced whether protonemas survived or died, and the highest recovery rate was observed at a very low RWC (0.3%) while protonemas with 100% RWC (i.e., fully hydrated) died regardless of age or recovery time.

### 3.5 Relative Water Content and Age: Impact on Freezing Tolerance of *S. caninervis*

**FIGURE 6:**
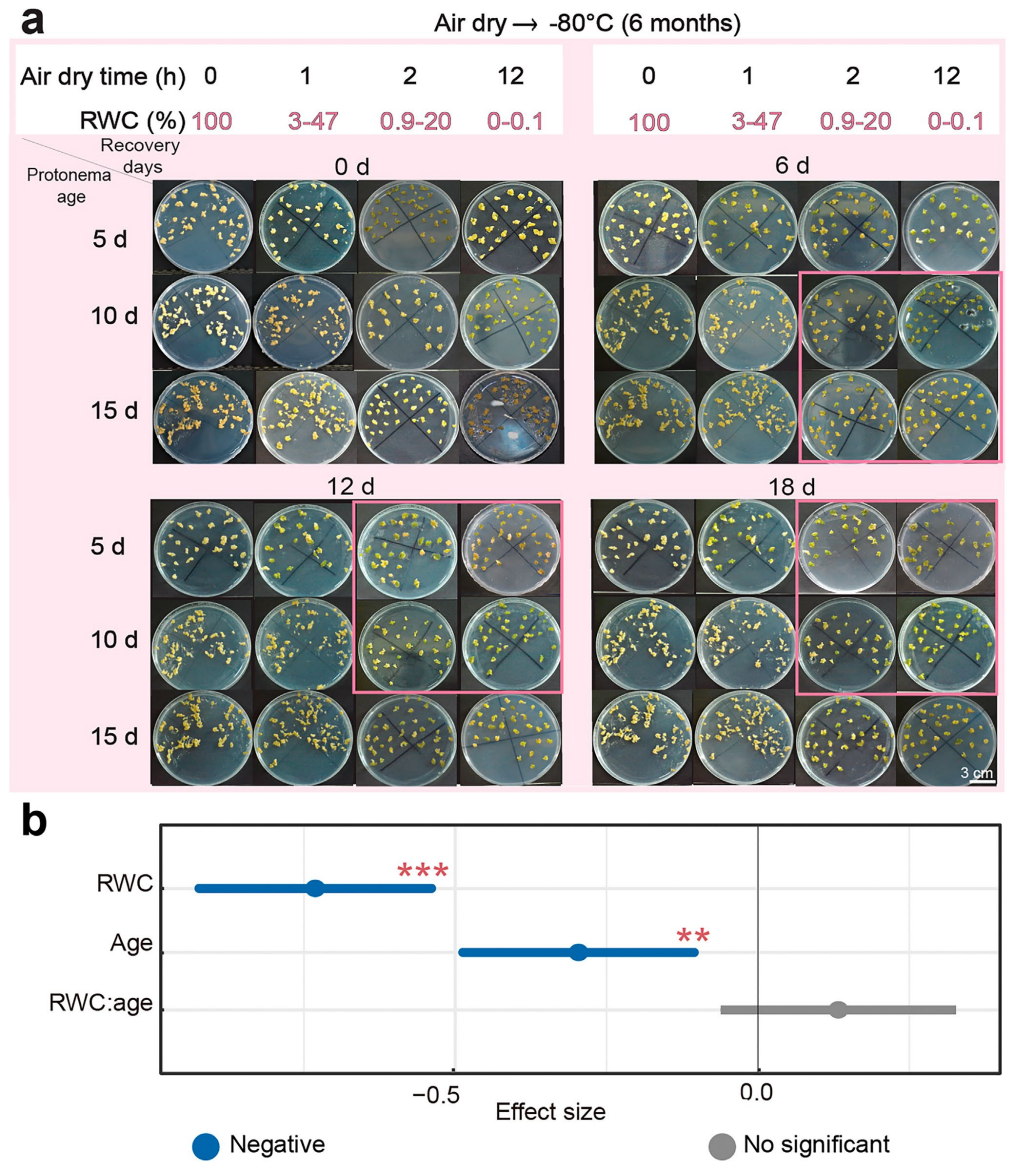
Growth recovery of *S. caninervis* protonemas on Knop's medium after freezing at an ultra‐low temperature. (a) In vitro growth on Petri dishes after different air‐drying times, and for three ages of protonemas. (b) Effect of RWC, age, and RWC and age on the recovery rate of protonemas using a linear mixed‐effects model. The recovery rate of protonemas was measured at 6‐day intervals starting from the day of culture. Protonemas were collected at ages of 5, 10, and 15 days and exposed to air‐drying for 0, 1, 2, and 12 h. The air‐dried protonemas were then stored at −80°C in an ultra‐low temperature freezer for 6 months. After storage, the protonemas were then transferred to fresh Knop's medium in Petri dishes and cultured in a growth chamber. Phenotypic observations were made at 0, 6, 12, and 18 days after recovery. Pink boxes highlight the highest recovery rates in response to freezing stress at −80°C. Data are presented as the mean ± SD of the estimated effect sizes. Statistical significance: **p < 0.01, and ***p < 0.001.

The freezing tolerance of *S. caninervis* protonemas was significantly influenced by age (5, 10, and 15 days), RWC corresponding to air‐drying times (0, 1, 2, and 12 h), and the duration of recovery (0, 6, 12, and 18 days) (Figure 6 and Table S4). The results indicate that RWC and age were the two major determinants of recovery rate in *S. caninervis* protonemas (Figure 6b). Fully hydrated (100% RWC) protonemas from all age groups (5, 10, and 15 days) did not recover. As RWC decreased, recovery rates increased. After 1 h of air drying (3.49% RWC), 5‐day‐old protonemas showed a 56.3% recovery rate, which increased to 74.26% and 100% after 2 h (0.9% RWC) and 12 h (0% RWC), respectively. After 1 h of air drying (24.5% RWC), no recovery was observed in 10‐day‐old protonemas. However, at 2 h (2.69% RWC) and 12 h (0.1% RWC), 77.26% and 95.5% recovery rates, respectively, were recorded. A similar trend was observed in 15‐day‐old protonemas, with no recovery after 1 h of air drying (47.12% RWC). Recovery rates of 31.33% and 44.3% were noted at 2 h (20.9% RWC) and 12 h (0.1% RWC), respectively (Figure S3).

Age also influenced the recovery rate, with only 5‐ and 10‐day‐old protonemas showing significant recovery (100% and 95.5%, respectively) when fully dehydrated (~0.1% RWC). In contrast, the lowest recovery rate (44.3%) was observed in 15‐day‐old protonemas when they were fully dehydrated (~0.1% RWC) (Figure S3 and Table S4). The response of *S. caninervis* protonemas to ultra‐low temperature was significantly influenced by RWC; moreover, the influence of RWC (effect size = −0.73) was greater than that of age (effect size = −0.30) (Figure 6b). The highest recovery rate was observed in young and fully dried protonemas, while fully hydrated (100% RWC) protonemas died, regardless of age.

## 4 Discussion

It is broadly acknowledged that mosses posses freezing tolerance. Previous research indicated that some mosses can survive freezing stress at −30°C without cellular damage even when desiccated (Dilks and Proctor 1975). Mosses have evolved mechainsm to withstand freezing temperatures without significant damage, enabling them to endure extreme stress (Charron and Quatrano 2009, Takezawa 2011, Turetsky et al. 2012, Roads and Longton 2014). Unlike the cells of vascular plants, moss cells have a thin wall barrier that allows water to move easily betweeen tissues and their surroudings allowing them to loose water rapidly, reducing the formation of intracellular ice crystals, protecting cells and enabling rapid water loss as a mechanism to cope with freezing stress (Scott 2002, Proctor et al. 2007, Lenné et al. 2010).

4.1

Our study revealed that RWC plays a critical role in determining the response of three moss species to freezing stresss (Figures 5b and 6b). Notably, the protonemas of *P. patens*, *B. aregenteum*, and *S*. *caninervis* exhibited similar pattern of RWC loss, characterized by a significant decrease in RWC with prolonged air‐drying times. Low RWC is vital to improving the tolerance of protonemas to ultra‐low temperatures and aiding recovery from harsh conditions. This study also demonstarted that *B. argenteum* and *S.caninervis* protonemas had the ability to tolerate ultra‐ low temperatures and recover significantly after undergoing dehydration for 12 h. This highlights the importance of RWC in maintaining cellular integrity under freezing stress (Figure 3). Young protonemas lose water faster than older protonemas, and this might be due to their smaller cells and thin filaments that allow water to escape quickly, whereas older protonemas have more rigid and well‐developed cell walls (Pressel and Duckett 2010). After 1 h of air drying, RWC dropped by 92.0%–96.51% (3.49%–8.0%), decreasing further to 97.5%–99.1% (0.9%–3.5%) after 2–12 h and eventually reaching nearly zero (0%–0.3%) (Figure 3). The protonemas of desiccation‐tolerant mosses like *Funaria hygrometrica* , *Campylopus introflexus* , *Syntrichia muralis*, and *P. patens* lose approximately 90% of their RWC after 2–3 days of slow drying or 30 min to 1 h of rapid drying (Proctor et al. 2007). RWC significantly influences mosses' ability to survive extremely low temperatures. The moss *Tortula ruralis* exhibited exceptional tolerance to extreme freezing in its desiccated state, retaining its capacity for protein and RNA synthesis even after exposure to temperatures as low as those of liquid nitrogen, so this remarkable resilience highlights the pivotal role of dehydration in conferring enhanced freezing tolerance in this species (Bewley 1973). In this study, fully hydrated protonemas of *P. patens* , *B. argenteum* , and *S. caninervis* showed no recovery, whereas *B. argenteum* and *S. caninervis* protonemas with reduced RWC showed significantly improved recovery rates. This also indicates that RWC is a key factor in the tolerance of these three moss species to freezing stress. The Antarctic moss *Polytrichum alpestre* was substantially susceptible to low temperatures and freeze–thaw cycles, with the majority of the dehydrated samples exhibiting a greater tolerance to freezing stress than hydrated ones (Kennedy 1993). Interestingly, *P. patens* showed no recovery even with desiccated protonemas, highlighting their limited ability to survive at extremely low temperatures (Figure 4). Previous studies revealed that pretreatment with abscisic acid (Begum et al. 2012) and cold acclimation (Minami et al. 2003, 2005) enhanced freezing resistance in *P. patens* protonemas. While *P. patens* thrives in temperate zones, commonly near lakes and rivers, and in moist environments (Cove 2005; Frank et al. 2005), both *B. argenteum* and *S. caninervis* demonstrated exceptional recovery rates when exposed to an ultra‐low temperature (Figures 5 and 6). *Bryum rubens* protonemas effectively survived cryopreservation, even without encapsulation, whereas only 20%–30% of *Ditrichum cornubicum* protonemas persisted, while *Cyclodictyon laetevirens* protonemas failed to survive dehydration and freezing stress (Pence 2008). This highlights the remarkable ability of *B. argenteum* and *S. caninervis* to freezing stress.

4.2

The age‐dependent response to freezing stress in these three moss species reveals a critical window of tolerance, with *B. argenteum* and *S. caninervis* exhibiting optimal recovery rates at specific developmental stages, suggesting that age and RWC interact to confer exceptional stress tolerance (Figures 5b and 6b). Under fully dried conditions, *P. patens* protonemas showed no recovery at any age, whereas *B. argenteum* protonemas exhibited significant recovery rates when they were 10 and 15 days old (99.6% and 94.4%, respectively). Similarly, *S. caninervis* protonemas (fully dried) showed recovery rates of 100% and 95.5% when they were 5 and 10 days old, respectively (Figures S2 and S3). These results imply that *B. argenteum* and *S. caninervis* exhibit exceptional stress tolerance in response to extremely low temperatures, which is most likely caused by the complex interplay between age and RWC. The resistance of mosses to freezing stress depends on factors such as species, age, tissue type, native habitat, water content, and seasonal variations (Minami et al. 2005; La Farge et al. 2013; Agurla et al. 2018). A comprehensive study revealed that mature leaves of *Mnium undulatum* possessed significantly greater frost tolerance than young leaves, attributed to their capacity for extracellular freezing and rapid dehydration, thereby preventing the formation of lethal intracellular ice (Hudson and Brustkern 1965).

Additionally, the variation in recovery rates of protonemas among the three moss species is likely due to their differences in biological traits and geographic distributions. *B. argenteum* is widely distributed globally (Biersma et al. 2018; Huttunen et al. 2018), and has also been detected in the Antarctic region (Pisa et al. 2014), which may explain its ability to acquire strong tolerance to freezing stress. Several mosses have developed essential characteristics that enable them to survive in extreme climates, such as broad responsiveness of net assimilation rates to temperature and a high degree of phenotypic flexibility (Turetsky et al. 2012). *S. caninervis* is primarily found in Asia's cold deserts (Wu et al. 2012; Tanaka et al. 2018; Coe et al. 2021; Ladrón De Guevara and Maestre 2022; Mao et al. 2022) and its gametophytes can survive at −80°C for 5 years (Li et al. 2024). Consistent with this, our study showed that *S. caninervis* exhibits adaptive mechanisms during freezing stress. Desert moss may alter the composition of its cell membranes by increasing the proportion of unsaturated fatty acids, which are essential for maintaining membrane fluidity and structure at lower temperatures, and this adjustment helps to stabilize the membranes, reducing the risk of damage caused by ice crystal formation (Ouellet and Charron 2013; Juurakko et al. 2021).

However, fully hydrated *S. caninervis* gametophytes can survive freezing treatment only at −16°C by regenerating many new branches (Bai et al. 2024). In response to low temperatures, *S. caninervis* accumulates a high level of sugar that allows it to maintain cell integrity and provide energy during rapid recovery from stress (Xu et al. 2009; Zhang and Zhang 2020). It also possesses a strong antioxidant system that allows it to combat stresses by suppressing the negative impact of reactive oxygen species (Salih et al. 2024). Tissues in bryophytes exhibit a characteristic that enables plants to endure freezing stress, namely an increase in the synthesis of cryoprotectants, such as proteins and sugars, allowing cells to maintain their integrity and serve as an energy source during the recovery period (Uemura et al. 2006). These findings highlight the critical role of membrane composition in the freezing tolerance of desert mosses, providing insight into their remarkable ability to survive in extreme environments (Li et al. 2024). The vulnerability of a living cell to intracellular freezing and tension‐induced cavitation during freeze/thaw events can be influenced by variations in wall stiffness (Takezawa 2011).

## 5 Conclusions

This study revealed that the protonemas of three moss species responded differently to an ultra‐low temperature. While *P. patens* protonemas did not survive exposure to −80°C, *B. argenteum* and *S. caninervis* protonemas exhibited significant recovery rates. The two key factors influencing those recovery rates were RWC and age. Younger protonemas, with undifferentiated cells, showed greater resilience, particularly those of *S. caninervis* . Young (5‐ and 10‐day‐old) and fully dehydrated *S. caninervis* protonemas achieved 98.6% and 95.5% recovery rates, respectively, compared to *B. argenteum* , which reached a maximum of 80.5% and 99.6%, respectively, for protonemas of these two ages. These findings provide a deeper understanding of resilience mechanisms in mosses, laying a foundation for future research on their adaptation strategies to freezing‐stress environments, including during cryopreservation.

## Ethics Statement

The authors have nothing to report.

## Consent

The authors have nothing to report.

## Conflicts of Interest

The authors declare no conflicts of interest.

## Supporting information


**Figure S1:** Growth of *P. patens* , *B. argenteum* , and *S. caninervis* protonemas on Knop's medium. (a) The developmental stage of protonemas from a single filament across different days of culture. (b) The area index of the growth of protonemas of the three moss species was measured on various days after culture. (c) The production of bud numbers in three moss species at early stages of development in protonemas (1, 3, 5, and 7 days old). Each moss is represented by a different color. Data were analyzed using two‐way ANOVA at a 95% CL. Significant differences compared to the control (0 h) were determined using the LSD multiple comparison test: *p < 0.05, **p < 0.01, and ***p < 0.001.
**Figure S2:** Regeneration rates of *B. argenteum* protonemas across various recovery days and time points after treatment at an ultra‐low temperature. The data are presented in three separate panels, each corresponding to different ages of protonemas (5, 10, and 15 days). The regeneration rates are expressed as percentages and are color‐coded to represent different time points: 0 h (100% RWC, white), 1 h (6%–50% RWC, light purple), 2 h (2%–23% RWC, pink), and 12 h (0%–0.3% RWC, dark purple). The data are expressed as the mean ± SD from three biological replicates. Data were analyzed using two‐way ANOVA at a 95% CL. Significant differences compared to the control (0 h) were determined using the LSD multiple comparison test: *p < 0.05, **p < 0.01, ***p < 0.001.
**Figure S3:**. Regeneration rates of *S. caninervis* protonemas across various recovery days and time points after treatment at an ultra‐low temperature. The data is presented in three separate panels, each corresponding to different ages of protonemas (5, 10, and 15 days). The regeneration rates are expressed as percentages and are color‐coded to represent different time points: 0 h (100% RWC, white), 1 h (3%–47% RWC, light blue), 2 h (0.9%–20% RWC, medium blue), and 12 h (0%–0.1% RWC, dark blue). The data are expressed as the mean ± SD from three biological replicates. Data were analyzed using two‐way ANOVA at a 95% CL. Significant differences compared to the control (0 h) were determined using the LSD multiple comparison test: *p < 0.05, **p < 0.01, ***p < 0.001.

**Table S1:** The treatment groups information in this study.
**Table S2:**. Differences in relative water content in the protonemas of three moss species.
**Table S3:** Recovery rates of *B. argentum* protonemas under freezing stress.
**Table S4:** Recovery rates of *S. caninervis* protonemas under freezing stress.
**Table S5:** Differences in the physiological parameters of the protonemas of three moss species.

## Data Availability

All related data are available within the manuscript and its additional files.
