# Supplementary material for: Outstanding Ultra‐Low Freezing Tolerance in Moss Species: Insights From Recovery Ability
Source: Plant Environ Interact. 2025 Sep 24;6(5):e70081. doi: 10.1002/pei3.70081 (PMC12460189; doi:10.1002/pei3.70081)
Supplement: Supplementary file 1 — Figure S1: Growth of P. patens , B. argenteum , and S. caninervis protonemas on Knop's medium. (a) The developmental stage of protonemas from a single filament across different days of culture. (b) The area index of the growth of protonemas of the three moss species was measured on various days after culture. (c) The production of bud numbers in three moss species at early stages of development in protonemas (1, 3, 5, and 7 days old). Each moss is represented by a different color. Data were analyzed using two‐way ANOVA at a 95% CL. Significant differences compared to the control (0 h) were determined using the LSD multiple comparison test: *p < 0.05, **p < 0.01, and ***p < 0.001. Figure S2: Regeneration rates of B. argenteum protonemas across various recovery days and time points after treatment at an ultra‐low temperature. The data are presented in three separate panels, each corresponding to different ages of protonemas (5, 10, and 15 days). The regeneration rates are expressed as percentages and are color‐coded to represent different time points: 0 h (100% RWC, white), 1 h (6%–50% RWC, light purple), 2 h (2%–23% RWC, pink), and 12 h (0%–0.3% RWC, dark purple). The data are expressed as the mean ± SD from three biological replicates. Data were analyzed using two‐way ANOVA at a 95% CL. Significant differences compared to the control (0 h) were determined using the LSD multiple comparison test: *p < 0.05, **p < 0.01, ***p < 0.001. Figure S3:. Regeneration rates of S. caninervis protonemas across various recovery days and time points after treatment at an ultra‐low temperature. The data is presented in three separate panels, each corresponding to different ages of protonemas (5, 10, and 15 days). The regeneration rates are expressed as percentages and are color‐coded to represent different time points: 0 h (100% RWC, white), 1 h (3%–47% RWC, light blue), 2 h (0.9%–20% RWC, medium blue), and 12 h (0%–0.1% RWC, dark blue). The data are expressed as the mean ± SD from t [file PEI3-6-e70081-s002.docx]

**Supplementary files**

**Fig. S1:** Growth of *P. patens*, *B. argenteum* and *S. caninervis* protonemas on Knop’s medium.

**Fig. S2:** Regeneration rates of *B. argenteum* protonemas across various recovery days and time points after treatment at an ultra-low temperature.

**Fig. S3:** Regeneration rates of *S. caninervis* protonemas across various recovery days and time points after treatment at an ultra-low temperature.

**Table S1**: Data of protonema age, air-drying, and freezing stress

**Table S2**: Differences in relative water content in the protonemas of three moss species

**Table S3:** Recovery percentage of *B. argentum* protonemas under freezing stress.

**Table S4:** Recovery percentage of *S. caninervis* protonemas under freezing stress

**Table S5:** Differences in the physiological parameters of the protonemas of three moss species


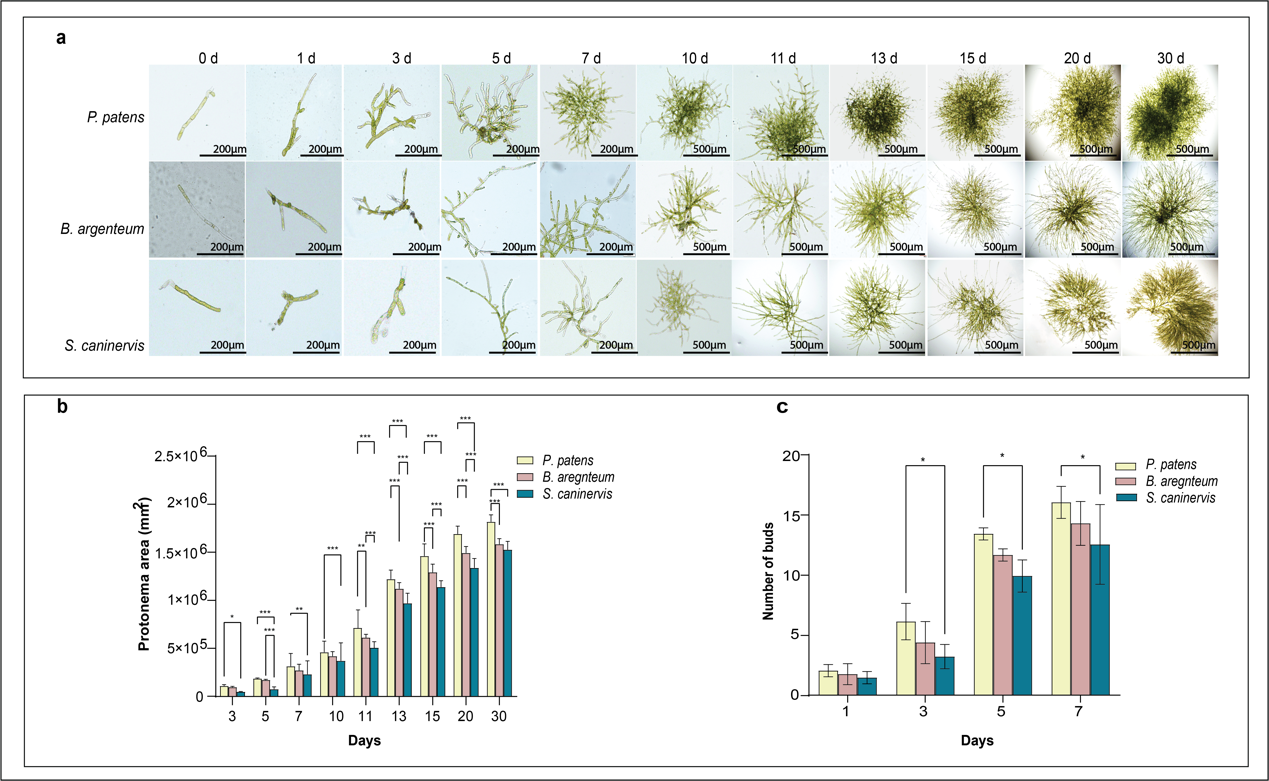


**Fig. S1.** **Growth of *P. patens*, *B. argenteum*, and *S. caninervis* protonemas on Knop’s medium**. (**a**) The developmental stage of protonemas from a single filament across different days of culture. (**b**) The area index of the growth of protonemas of the three moss species was measured on various days after culture. (**c**) The production of bud numbers in three moss species at early stages of development in protonemas (1, 3, 5 and 7 days old). Each moss is represented by a different color. Data were analyzed using two-way ANOVA at a 95% CL Significant differences compared to the control (0 h) were determined using the LSD multiple comparison test: * *p* < 0.05, ** *p* < 0.01, *** *p* < 0.001.


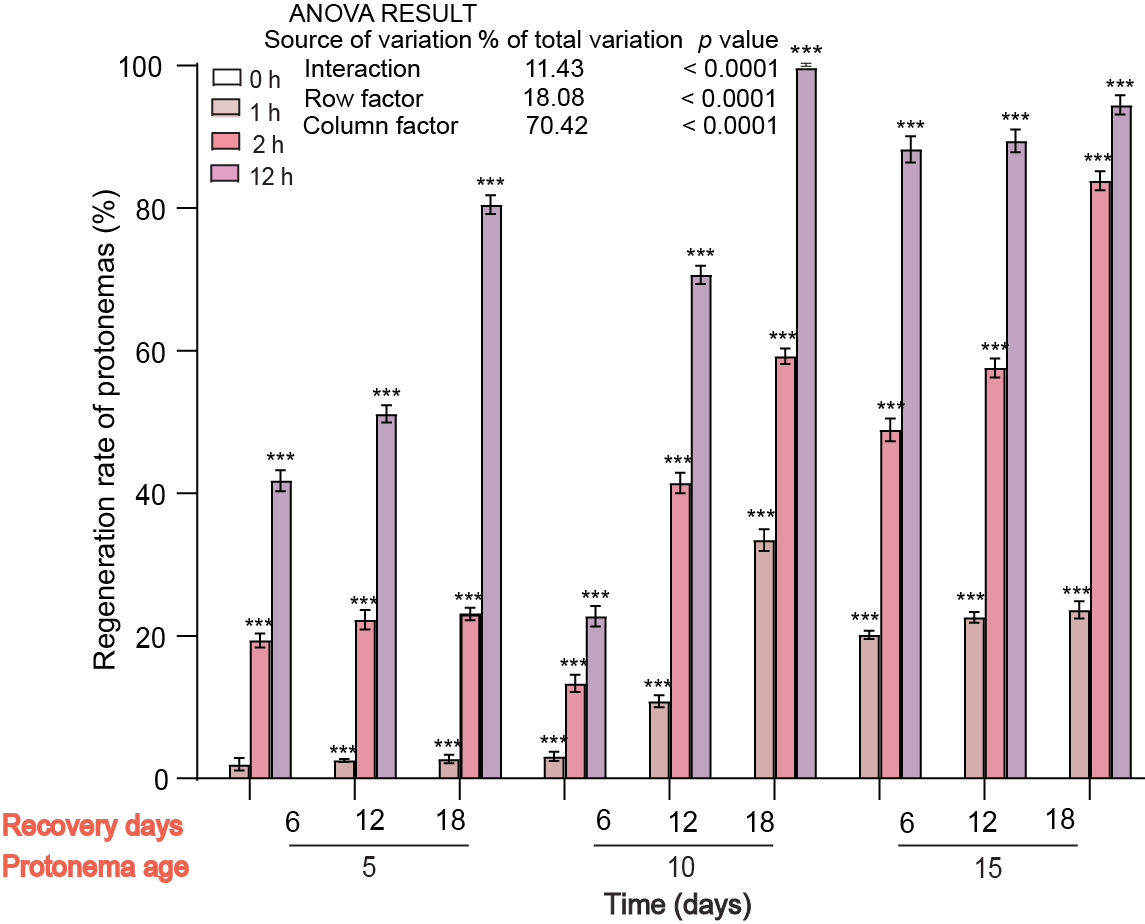


**Fig. S2.** **Regeneration rates of *B. argenteum* protonemas across various recovery days and time points after treatment** **at an ultra-low temperature.** The data is presented in three separate panels, each corresponding to different ages of protonemas (5, 10 and 15 days). The regeneration rates are expressed as percentages and are color-coded to represent different time points: 0 h (100% RWC, white), 1 h (6–50% RWC, light purple), 2 h (2–23% RWC, pink), and 12 h (0–0.3% RWC, dark purple). The data are expressed as the mean ± SD from three biological replicates. Data were analyzed using two-way ANOVA at a 95% CL Significant differences compared to the control (0 h) were determined using the LSD multiple comparison test: * *p* < 0.05, ** *p* < 0.01, *** *p* < 0.001.


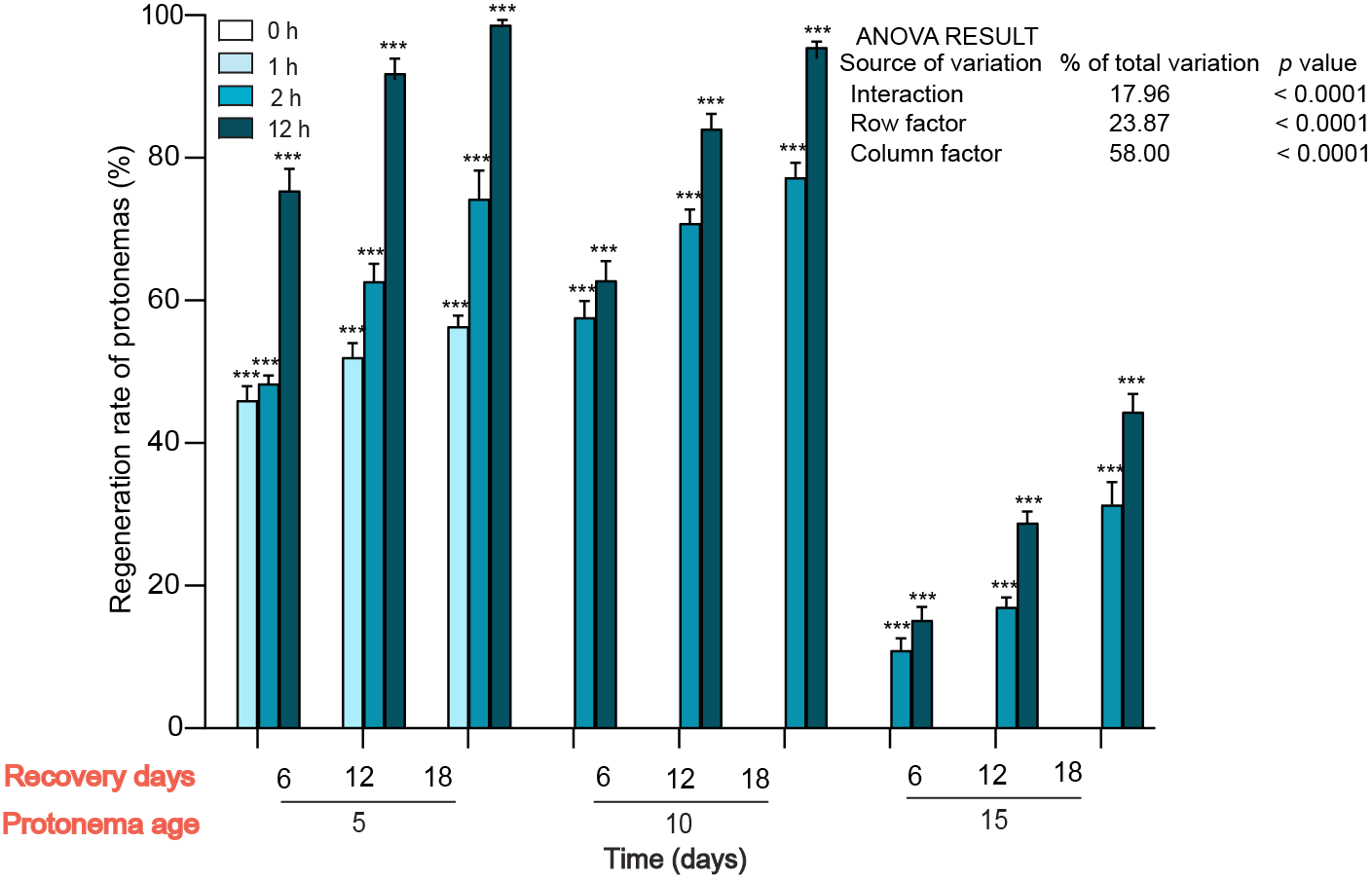


**Fig. S3. Regeneration rates of *S. caninervis* protonemas across various recovery days and time points after treatment at an ultra-low temperature.** The data is presented in three separate panels, each corresponding to different ages of protonemas (5, 10 and 15 days). The regeneration rates are expressed as percentages and are color-coded to represent different time points: 0 h (100% RWC, white), 1 h (3–47% RWC, light blue), 2 h (0.9–20% RWC, medium blue), and 12 h (0–0.1% RWC, dark blue). The data are expressed as the mean ± SD from three biological replicates. Data were analyzed using two-way ANOVA at a 95% CL Significant differences compared to the control (0 h) were determined using the LSD multiple comparison test: * *p* < 0.05, ** *p* < 0.01, *** *p* < 0.001.

**Table S1**: Data of protonema age, air-drying, and freezing stress

| **Specie name** | **Age of protonemas (days)** | **Air-drying time (h)** | **Storage** |
| --- | --- | --- | --- |
| *P. patens* | 5 | 0 | -80°C |
|  |  | 1 |  |
|  |  | 2 |  |
|  |  | 12 |  |
|  | 10 | 0 |  |
|  |  | 1 |  |
|  |  | 2 |  |
|  |  | 12 |  |
|  | 15 | 0 |  |
|  |  | 1 |  |
|  |  | 2 |  |
|  |  | 12 |  |
| *B. argenteum* | 5 | 0 | -80°C |
|  |  | 1 |  |
|  |  | 2 |  |
|  |  | 12 |  |
|  | 10 | 0 |  |
|  |  | 1 |  |
|  |  | 2 |  |
|  |  | 12 |  |
|  | 15 | 0 |  |
|  |  | 1 |  |
|  |  | 2 |  |
|  |  | 12 |  |
| *S. caninervis* | 5 | 0 | -80°C |
|  |  | 1 |  |
|  |  | 2 |  |
|  |  | 12 |  |
|  | 10 | 0 |  |
|  |  | 1 |  |
|  |  | 2 |  |
|  |  | 12 |  |
|  | 15 | 0 |  |
|  |  | 1 |  |
|  |  | 2 |  |
|  |  | 12 |  |

**Table S2**: Differences in relative water content in the protonemas of three moss species

| Species | Age | 0 h | 1 h | 2 h | 12 h |
| --- | --- | --- | --- | --- | --- |
| *P. patens* | 5 d | 100±0 | 8.0±0.3^***^ | 3.5±1.1^***^ | 0.3±0.6^***^ |
|  | 10 d | 100±0 | 38.5±3.9^**^ | 14.2±2.8^***^ | 0.3±0.3^***^ |
|  | 15 d | 100±0 | 53.6±2.3^***^ | 25.3±5.6^**^ | 13.4±1.2^***^ |
| *B. argenteum* | 5 d | 100±0 | 6.6±1.2^***^ | 2.2±1.9^**^ | 0.0±0.0^***^ |
|  | 10 d | 100±0 | 33.7±3.9^***^ | 11.2±3.0^***^ | 0.0±0.0^***^ |
|  | 15 d | 100±0 | 50.6±8.4^**^ | 23.6±7.1^**^ | 0.9±0.3^***^ |
| *S. caninervis* | 5 d | 100±0 | 3.4±2.0^***^ | 0.9±1.2^***^ | 0±0^***^ |
|  | 10 d | 100±0 | 24.5±4.7^**^ | 2.6±0.6^***^ | 0±0^***^ |
|  | 15 d | 100±0 | 47.12±1.1^***^ | 20.9±3.4^***^ | 0.19±0.3^***^ |

LSD multiple comparison test: **p* < 0.05, ***p* < 0.01, and ****p* < 0.001.

|  |  | **0 h** | | | | **1 h** | | | | **2 h** | | | | **12 h** | | | |
| --- | --- | --- | --- | --- | --- | --- | --- | --- | --- | --- | --- | --- | --- | --- | --- | --- | --- |
| **Age** | **Days** | **R1** | **R2** | **R3** | **Mean±SD** | **R1** | **R2** | **R3** | **Mean±SD** | **R1** | **R2** | **R3** | **Mean±SD** | **R1** | **R2** | **R3** | **Mean±SD** |
| 5 | 6 | 0 | 0 | 0 | 0±0 | 2.8 | 1.06 | 2 | 1.9±0.8 | 18.21 | 20.1 | 19.76 | 19.3±1.0 | 43.1 | 42.05 | 40.2 | 41.7±1.4 |
|  | 12 | 0 | 0 | 0 | 0±0 | 2.35 | 2.5 | 2.75 | 2.5±0.2 | 21 | 23.7 | 22.1 | 22.2±1.3 | 52.273 | 51.3 | 49.86 | 51.1±1.2 |
|  | 18 | 0 | 0 | 0 | 0±0 | 2.3 | 3.4 | 2.5 | 2.7±0.5 | 22.24 | 23 | 24 | 23.0±0.8 | 79.01 | 81.5 | 81 | 80.5±1.3 |
| 10 | 6 | 0 | 0 | 0 | 0±0 | 3.8 | 3 | 2.47 | 3.0±0.6 | 14.29 | 13.7 | 12 | 13.3±1.1 | 21.21 | 24 | 23 | 22.7±1.4 |
|  | 12 | 0 | 0 | 0 | 0±0 | 11.54 | 11 | 9.9 | 10.8±0.8 | 42.86 | 40 | 41.5 | 41.4±1.4 | 70.43 | 69.5 | 72 | 70.6±1.2 |
|  | 18 | 0 | 0 | 0 | 0±0 | 34.62 | 31.7 | 34 | 33.4±1.5 | 60 | 58 | 59.7 | 59.2±1.0 | 100 | 100 | 100 | 99.6±0.2 |
| 15 | 6 | 0 | 0 | 0 | 0±0 | 19.5 | 20.47 | 20.45 | 20.1±0.5 | 50 | 47.1 | 49.65 | 48.9±1.5 | 88.03 | 86.56 | 90.2 | 88.2±1.8 |
|  | 12 | 0 | 0 | 0 | 0±0 | 23.07 | 21.7 | 23 | 22.5±0.7 | 59.143 | 57 | 56.6 | 57.5±1.3 | 90.7 | 87.65 | 90 | 89.4±1.5 |
|  | 18 | 0 | 0 | 0 | 0±0 | 23 | 22.9 | 25 | 23.6±1.1 | 85.33 | 83.5 | 82.75 | 83.6±1.3 | 93.45 | 94 | 96 | 94.4±1.3 |

**Table S3:** Recovery percentage of *B. argentum* protonemas under freezing stress

**Table S4:** Recovery percentage of *S. caninervis* protonemas under freezing stress

|  | **0 h** | | | | | **1 h** | | | | **2 h** | | | | **12 h** | | | |
| --- | --- | --- | --- | --- | --- | --- | --- | --- | --- | --- | --- | --- | --- | --- | --- | --- | --- |
| **Age** | **Days** | **R1** | **R2** | **R3** | **Mean±SD** | **R1** | **R2** | **R3** | **Mean±SD** | **R1** | **R2** | **R3** | **Mean±SD** | **R1** | **R2** | **R3** | **Mean±SD** |
| 5 | 6 | 0 | 0 | 0 | 0±0 | 48 | 46 | 44 | 46±2 | 49 | 47 | 49 | 48.3±1.1 | 76.1905 | 72 | 78 | 75.3±3.0 |
|  | 12 | 0 | 0 | 0 | 0±0 | 52 | 54 | 50 | 52±2 | 62.96 | 65 | 60 | 62.6±2.5 | 90.4764 | 95 | 90 | 91.8±2.7 |
|  | 18 | 0 | 0 | 0 | 0±0 | 56 | 55 | 58 | 56.3±1.5 | 77.78 | 70 | 75 | 74.2±3.9 | 99 | 98 | 99 | 98.6±0.5 |
| 10 | 6 | 0 | 0 | 0 | 0±0 | 0 | 0 | 0 | 0±0 | 58.89 | 55 | 59 | 57.6±2.2 | 63.6207 | 59.7 | 65 | 62.7±2.7 |
|  | 12 | 0 | 0 | 0 | 0±0 | 0 | 0 | 0 | 0±0 | 69.44 | 73 | 70 | 70.8±1.9 | 86.2068 | 82 | 84 | 84.0±2.1 |
|  | 18 | 0 | 0 | 0 | 0±0 | 0 | 0 | 0 | 0±0 | 77.78 | 79 | 75 | 77.2±2.0 | 96.55 | 97 | 93 | 95.5±2. |
| 15 | 6 | 0 | 0 | 0 | 0±0 | 0 | 0 | 0 | 0±0 | 11.79 | 9 | 12 | 10.9±1.6 | 16.67 | 13 | 15.7 | 15.1±1.9 |
|  | 12 | 0 | 0 | 0 | 0±0 | 0 | 0 | 0 | 0±0 | 16.67 | 15.9 | 18.5 | 17.02±1.3 | 29.41 | 30 | 27 | 28.8±1.5 |
|  | 18 | 0 | 0 | 0 | 0±0 | 0 | 0 | 0 | 0±0 | 30 | 29 | 35 | 31.3±3.2 | 44.12 | 47 | 42 | 44.3±2.5 |

**Table S5:** Differences in the physiological parameters of the protonemas of three moss species

| Specie Name | Area of protonemas (mm^2^) | Number of buds per protonema | Chl *a* | Chl *b* | Total Chl |
| --- | --- | --- | --- | --- | --- |
| *P. patens* | 1.0±0.7×10^6͘͘*^ | 10.8±7.4 | 8.8±1.5 | 4.3±1.2 | 13.1±2.6 |
| *B. argenteum* | 9.4±0.6×10^6*^ | 9.2±6.8 | 7.7±1.4 | 3.9±1.2 | 11.7±2.4 |
| *S. caninervis* | 8.3±0.6×10^6***^ | 7.6±6.1^*^ | 6.0±1.8^**^ | 3.5±1.0 | 9.6±2.5^***^ |

LSD multiple comparison test: **p* < 0.05, ***p* < 0.01, and ****p* < 0.001.
