# Supplementary material for: Outstanding Ultra‐Low Freezing Tolerance in Moss Species: Insights From Recovery Ability
Source: Plant Environ Interact. 2025 Sep 24;6(5):e70081. doi: 10.1002/pei3.70081 (PMC12460189; doi:10.1002/pei3.70081)
Supplement: Supplementary file 2 — Table S1: The treatment groups information in this study. Table S2:. Differences in relative water content in the protonemas of three moss species. Table S3: Recovery rates of B. argentum protonemas under freezing stress. Table S4: Recovery rates of S. caninervis protonemas under freezing stress. Table S5: Differences in the physiological parameters of the protonemas of three moss species. [file PEI3-6-e70081-s001.docx]

**Table S1**: Treatment groups information in this study

| **Specie name** | **Age of protonemas (days)** | **Air-drying time (h)** | **Storage** |
| --- | --- | --- | --- |
| *P. patens* | 5 | 0 | -80°C |
|  |  | 1 |  |
|  |  | 2 |  |
|  |  | 12 |  |
|  | 10 | 0 |  |
|  |  | 1 |  |
|  |  | 2 |  |
|  |  | 12 |  |
|  | 15 | 0 |  |
|  |  | 1 |  |
|  |  | 2 |  |
|  |  | 12 |  |
| *B. argenteum* | 5 | 0 | -80°C |
|  |  | 1 |  |
|  |  | 2 |  |
|  |  | 12 |  |
|  | 10 | 0 |  |
|  |  | 1 |  |
|  |  | 2 |  |
|  |  | 12 |  |
|  | 15 | 0 |  |
|  |  | 1 |  |
|  |  | 2 |  |
|  |  | 12 |  |
| *S. caninervis* | 5 | 0 | -80°C |
|  |  | 1 |  |
|  |  | 2 |  |
|  |  | 12 |  |
|  | 10 | 0 |  |
|  |  | 1 |  |
|  |  | 2 |  |
|  |  | 12 |  |
|  | 15 | 0 |  |
|  |  | 1 |  |
|  |  | 2 |  |
|  |  | 12 |  |

**Table S2**: Differences in relative water content in the protonemas of three moss species

| Species | Age | 0 h | 1 h | 2 h | 12 h |
| --- | --- | --- | --- | --- | --- |
| *P. patens* | 5 d | 100±0 | 8.0±0.3^***^ | 3.5±1.1^***^ | 0.3±0.6^***^ |
|  | 10 d | 100±0 | 38.5±3.9^**^ | 14.2±2.8^***^ | 0.3±0.3^***^ |
|  | 15 d | 100±0 | 53.6±2.3^***^ | 25.3±5.6^**^ | 13.4±1.2^***^ |
| *B. argenteum* | 5 d | 100±0 | 6.6±1.2^***^ | 2.2±1.9^**^ | 0.0±0.0^***^ |
|  | 10 d | 100±0 | 33.7±3.9^***^ | 11.2±3.0^***^ | 0.0±0.0^***^ |
|  | 15 d | 100±0 | 50.6±8.4^**^ | 23.6±7.1^**^ | 0.9±0.3^***^ |
| *S. caninervis* | 5 d | 100±0 | 3.4±2.0^***^ | 0.9±1.2^***^ | 0±0^***^ |
|  | 10 d | 100±0 | 24.5±4.7^**^ | 2.6±0.6^***^ | 0±0^***^ |
|  | 15 d | 100±0 | 47.12±1.1^***^ | 20.9±3.4^***^ | 0.19±0.3^***^ |

LSD multiple comparison test: **p* < 0.05, ***p* < 0.01, and ****p* < 0.001.

|  |  | **0 h** | | | | **1 h** | | | | **2 h** | | | | **12 h** | | | |
| --- | --- | --- | --- | --- | --- | --- | --- | --- | --- | --- | --- | --- | --- | --- | --- | --- | --- |
| **Age** | **Days** | **R1** | **R2** | **R3** | **Mean±SD** | **R1** | **R2** | **R3** | **Mean±SD** | **R1** | **R2** | **R3** | **Mean±SD** | **R1** | **R2** | **R3** | **Mean±SD** |
| 5 | 6 | 0 | 0 | 0 | 0±0 | 2.8 | 1.06 | 2 | 1.9±0.8 | 18.21 | 20.1 | 19.76 | 19.3±1.0 | 43.1 | 42.05 | 40.2 | 41.7±1.4 |
|  | 12 | 0 | 0 | 0 | 0±0 | 2.35 | 2.5 | 2.75 | 2.5±0.2 | 21 | 23.7 | 22.1 | 22.2±1.3 | 52.273 | 51.3 | 49.86 | 51.1±1.2 |
|  | 18 | 0 | 0 | 0 | 0±0 | 2.3 | 3.4 | 2.5 | 2.7±0.5 | 22.24 | 23 | 24 | 23.0±0.8 | 79.01 | 81.5 | 81 | 80.5±1.3 |
| 10 | 6 | 0 | 0 | 0 | 0±0 | 3.8 | 3 | 2.47 | 3.0±0.6 | 14.29 | 13.7 | 12 | 13.3±1.1 | 21.21 | 24 | 23 | 22.7±1.4 |
|  | 12 | 0 | 0 | 0 | 0±0 | 11.54 | 11 | 9.9 | 10.8±0.8 | 42.86 | 40 | 41.5 | 41.4±1.4 | 70.43 | 69.5 | 72 | 70.6±1.2 |
|  | 18 | 0 | 0 | 0 | 0±0 | 34.62 | 31.7 | 34 | 33.4±1.5 | 60 | 58 | 59.7 | 59.2±1.0 | 100 | 100 | 100 | 99.6±0.2 |
| 15 | 6 | 0 | 0 | 0 | 0±0 | 19.5 | 20.47 | 20.45 | 20.1±0.5 | 50 | 47.1 | 49.65 | 48.9±1.5 | 88.03 | 86.56 | 90.2 | 88.2±1.8 |
|  | 12 | 0 | 0 | 0 | 0±0 | 23.07 | 21.7 | 23 | 22.5±0.7 | 59.143 | 57 | 56.6 | 57.5±1.3 | 90.7 | 87.65 | 90 | 89.4±1.5 |
|  | 18 | 0 | 0 | 0 | 0±0 | 23 | 22.9 | 25 | 23.6±1.1 | 85.33 | 83.5 | 82.75 | 83.6±1.3 | 93.45 | 94 | 96 | 94.4±1.3 |

**Table S3:** Recovery rates of *B. argentum* protonemas under freezing stress

**Table S4:** Recovery rates of *S. caninervis* protonemas under freezing stress

|  | **0 h** | | | | | **1 h** | | | | **2 h** | | | | **12 h** | | | |
| --- | --- | --- | --- | --- | --- | --- | --- | --- | --- | --- | --- | --- | --- | --- | --- | --- | --- |
| **Age** | **Days** | **R1** | **R2** | **R3** | **Mean±SD** | **R1** | **R2** | **R3** | **Mean±SD** | **R1** | **R2** | **R3** | **Mean±SD** | **R1** | **R2** | **R3** | **Mean±SD** |
| 5 | 6 | 0 | 0 | 0 | 0±0 | 48 | 46 | 44 | 46±2 | 49 | 47 | 49 | 48.3±1.1 | 76.1905 | 72 | 78 | 75.3±3.0 |
|  | 12 | 0 | 0 | 0 | 0±0 | 52 | 54 | 50 | 52±2 | 62.96 | 65 | 60 | 62.6±2.5 | 90.4764 | 95 | 90 | 91.8±2.7 |
|  | 18 | 0 | 0 | 0 | 0±0 | 56 | 55 | 58 | 56.3±1.5 | 77.78 | 70 | 75 | 74.2±3.9 | 99 | 98 | 99 | 98.6±0.5 |
| 10 | 6 | 0 | 0 | 0 | 0±0 | 0 | 0 | 0 | 0±0 | 58.89 | 55 | 59 | 57.6±2.2 | 63.6207 | 59.7 | 65 | 62.7±2.7 |
|  | 12 | 0 | 0 | 0 | 0±0 | 0 | 0 | 0 | 0±0 | 69.44 | 73 | 70 | 70.8±1.9 | 86.2068 | 82 | 84 | 84.0±2.1 |
|  | 18 | 0 | 0 | 0 | 0±0 | 0 | 0 | 0 | 0±0 | 77.78 | 79 | 75 | 77.2±2.0 | 96.55 | 97 | 93 | 95.5±2. |
| 15 | 6 | 0 | 0 | 0 | 0±0 | 0 | 0 | 0 | 0±0 | 11.79 | 9 | 12 | 10.9±1.6 | 16.67 | 13 | 15.7 | 15.1±1.9 |
|  | 12 | 0 | 0 | 0 | 0±0 | 0 | 0 | 0 | 0±0 | 16.67 | 15.9 | 18.5 | 17.02±1.3 | 29.41 | 30 | 27 | 28.8±1.5 |
|  | 18 | 0 | 0 | 0 | 0±0 | 0 | 0 | 0 | 0±0 | 30 | 29 | 35 | 31.3±3.2 | 44.12 | 47 | 42 | 44.3±2.5 |

**Table S5:** Differences in the physiological parameters of the protonemas of three moss species

| Specie Name | Area of protonemas (mm^2^) | Number of buds per protonema | Chl *a* | Chl *b* | Total Chl |
| --- | --- | --- | --- | --- | --- |
| *P. patens* | 1.0±0.7×10^6͘͘*^ | 10.8±7.4 | 8.8±1.5 | 4.3±1.2 | 13.1±2.6 |
| *B. argenteum* | 9.4±0.6×10^6*^ | 9.2±6.8 | 7.7±1.4 | 3.9±1.2 | 11.7±2.4 |
| *S. caninervis* | 8.3±0.6×10^6***^ | 7.6±6.1^*^ | 6.0±1.8^**^ | 3.5±1.0 | 9.6±2.5^***^ |

LSD multiple comparison test: **p* < 0.05, ***p* < 0.01, and ****p* < 0.001.
